# CRISPR-Mediated Endogenous Activation of Fibroin Heavy Chain Gene Triggers Cellular Stress Responses in *Bombyx mori* Embryonic Cells

**DOI:** 10.3390/insects12060552

**Published:** 2021-06-13

**Authors:** Wenbo Hu, Xiaogang Wang, Sanyuan Ma, Zhangchuan Peng, Yang Cao, Qingyou Xia

**Affiliations:** 1 State Key Laboratory of Silkworm Genome Biology, Biological Science Research Center, Southwest University, Chongqing 400715, China; huwenbo@hotmail.com (W.H.); wangyang217804@126.com (X.W.); masy@swu.edu.cn (S.M.); sqdpzc@163.com (Z.P.); caoyang0458@swu.edu.cn (Y.C.); 2 Chongqing Key Laboratory of Sericulture Science, Chongqing Engineering and Technology Research Center for Novel Silk Materials, Chongqing 400715, China

**Keywords:** *Bombyx mori*, CRISPR, dCas9-VPR, cellular stress response, BmE cells

## Abstract

**Simple Summary:**

Based on a CRISPRa approach, activating endogenous fibroin heavy chain (*FibH*) gene expression in *Bombyx mori* embryonic (BmE) cells, which was driven by a combination of the dCas9-VPR (a tripartite activator, composed of VP64, p65, and Rta) and the sgRNA targeting to the promoter of *FibH* gene, was performed for investigating the biological roles of *FibH* in the development of silk gland cells. The activation of the endogenous *FibH* gene lead to up-regulation of cellular stress responses-related genes, which suggested a significant positive correlation between activated *FibH* gene expression and cellular stress responses. Moreover, the present findings might provide a potential model for studying the cellular stress responses caused by silk secretion disorder and lay a foundation for the understanding of silk gland development in silk-spinning insects.

**Abstract:**

The silkworm *Bombyx mori* is an economically important insect, as it is the main producer of silk. Fibroin heavy chain (*FibH*) gene, encoding the core component of silk protein, is specifically and highly expressed in silk gland cells but not in the other cells. Although the silkworm *FibH* gene has been well studied in transcriptional regulation, its biological functions in the development of silk gland cells remain elusive. In this study, we constructed a CRISPRa system to activate the endogenous transcription of *FibH* in *Bombyx mori* embryonic (BmE) cells, and the mRNA expression of *FibH* was successfully activated. In addition, we found that *FibH* expression was increased to a maximum at 60 h after transient transfection of sgRNA/dCas9-VPR at a molar ratio of 9:1. The qRT-PCR analysis showed that the expression levels of cellular stress response-related genes were significantly up-regulated along with activated *FibH* gene. Moreover, the lyso-tracker red and monodansylcadaverine (MDC) staining assays revealed an apparent appearance of autophagy in *FibH*-activated BmE cells. Therefore, we conclude that the activation of *FibH* gene leads to up-regulation of cellular stress responses-related genes in BmE cells, which is essential for understanding silk gland development and the fibroin secretion process in *B. mori*.

## 1. Introduction

The clustered regularly interspaced short palindromic repeats (CRISPR)/CRISPR-associated protein 9 (Cas9), a bacterial adaptive immune system, has been harnessed as a highly efficient genome editing platform and widely used to generate target mutations in an increasing number of organisms, including bacteria [1,2], plant [3,4,5], insect [6,7,8], zebrafish [9,10], and human [11,12,13]. In our previous studies, the CRISPR/Cas9 was successfully used as a genome editing tool in both *Bombyx mori* (*B. mori*) individuals and cultured cells [14,15,16]. Meanwhile, the dCas9, a mutant of Cas9 lacking detectable nuclease activity, has been developed to interfere or activate the gene expression instead of complete knockout, by fusing to a transcriptional repressive or activating domain [17]. The VPR activating domain is a tripartite activator composed of the VP64, p65, and Rta activators, which has also been fused to dCas9 [18]. Our previous work indicated that dCas9-VPR is sufficient to activate the expression of target genes in *B. mori* embryonic (BmE) cells [19].

The silkworm *B. mori* is an economically important insect, as it is the main producer of silk and a model organism for lepidopterans [20]. The silk gland is the unique organ for producing, storing, and processing silk fibers. The development of silk glands and the synthesis of silk proteins are two important processes to study in silkworm [21,22,23]. Silkworm cocoons are made of sericins and fibroins [23,24]. The sericins are a family of adhesive proteins that account for 25–30% weight of a cocoon, the rest are fibroins. The fibroin unit consists of six sets of disulfide-linked fibroin heavy (FibH, 350 kDa) and light (FibL, 25 kDa) chains with a fibrohexamerin protein (P25, 30 kDa) at a molar ratio of 6:6:1 [25,26]. Additionally, studies have revealed that the formation of disulfide linkage between FibH and FibL is important for the efficient secretion of fibroin from silk gland cells into the lumen [27,28,29]. Additionally, once the FibH fails to form a complete fibroin unit, it cannot be secreted out of the cells, and then leads to a silk gland atrophy [26,30].

*B. mori* fibroin-deficient mutants are valuable resources for studying the process of fibroin synthesis and secretion in the silk gland [20]. In a previous study, we found that deficient FibH perturbed the cellular homeostasis and caused posterior silk gland (PSG) cell atrophy in the fibroin-deficient naked pupa (*Nd*) mutant [31,32]. However, the molecular mechanism remains unclear. The *FibH* is a single copy gene in the *B. mori* genome, and over 90% of its encoding region is a highly repetitive GC-rich sequence, making it extremely hard to clone and to implement transgenic manipulation. The expression of *FibH* gene was completely closed in normal BmE cells. In this study, we used the CRISPRa system to specifically activate *FibH* gene expression in BmE cells. Then, we investigated the cellular stress responses caused by the ectopic activation of *FibH* through qRT-PCR and cell staining. The results show that cellular stress responses were significantly triggered in the *FibH*-activated BmE cells, which provided novel insight into the understanding of silk gland development and silk secretion process in silkworm *B. mori*.

## 2. Materials and Methods

### 2.1. Vector Construction

The dCas9 expression vector (pUC57-hr3/A4-dCas9) and sgRNA backbone vector (pUC57-U6-sgRNA) were produced by our laboratory. Codon-optimized effector domain VPR (1602 bp) was synthesized and inserted into pUC57-T-simple by a commercial service (Genescript, Nanjing, China). Transcriptional activator VPR was then fused to the C-terminal of dCas9 (4194 bp) via *Sph*I and *Hind*III digestion and T4 ligation as described previously [18,19,33]. The dCas9-VPR was driven by an hr3/A4 promoter (1711 bp) to construct the pUC57-A4-dCas9-VPR expression vector (8002 bp). Three sgRNA targeting the *FibH* promoter and a negative control sgRNA targeting an *EGFP* exon were synthesized as oligomers, then annealed and cloned into the sgRNA expression vector pUC57-U6-sgRNA (3678 bp) by using *Bbs*I digestion and T4 ligation. Sequences of sgRNAs are listed in Table S1.

### 2.2. Cell Transfection

BmE is a *B. mori* embryonic cell line [34] cultured in Grace medium supplemented with 10% fetal bovine serum at 27 °C. pUC57-A4-dCas9-VPR and pUC57-U6-sgRNA expression vectors (a total of 2.0 μg plasmids) were co-transfected into BmE cells, at a density of 2 × 10^5^ cells/well, using X-treme GENE Transfection Reagent (Roche, Basel, Switzerland).

### 2.3. Western Blot Assay

Forty-eight hours after transfection, proteins were extracted from BmE cells with NP-40 lysis buffer (Beyotime, Shanghai, China) containing the protease inhibitor phenylmethanesulfonyl fluoride (PMSF). The protein concentration was measured using the BCA assay (Beyotime, Shanghai, China). Protein samples (20 μg) were separated using 8% sodium dodecyl sulfate-polyacrylamide gel electrophoresis (SDS-PAGE) protein gel, and then transferred to a polyvinylidene fluoride (PVDF) membrane (GE Healthcare, Pittsburgh, USA). Subsequently, samples were treated with primary antibodies (Cas9/tubulin-specific antibody, 1:10,000) for 120 min at 37 °C before incubation for 60 min at 37 °C with a horseradish peroxidase (HRP)-coupled secondary sheep anti-mouse IgG antibody (1:20,000). Finally, the signal was detected with Pierce enhanced chemiluminescence (ECL) reagents (Thermo Fisher Scientific, New York, NY, USA).

### 2.4. Total RNA Extraction

Total RNA was extracted from the BmE cells using TRIzol reagent (Invitrogen, Carlsbad, CA, USA). Reverse transcription was performed on total RNA (1 μg) using a random primer (N6), an oligo (dT) primer, and the PrimeScript RT reagent Kit using gDNA Eraser (Takara, Kyoto, Japan) according to the manufacturer’s protocols.

### 2.5. Quantitative RT-PCR

The qRT-PCR was performed using SYBR *Premix Ex Taq* II (Takara, Kyoto, Japan) according to the manufacturer’s protocols and a qTOWER 2.0 real-time PCR system (Analytik Jena, Jena, Germany). The silkworm housekeeping gene *ribosomal protein L3* (*Rpl3*: GenBank accession number NM_001043661.1) was used as the internal control for RNA normalization. Primer sets for qRT-PCR are listed in Table S2. Three independent replicates were analyzed.

### 2.6. Autophagy Assay

Cell autophagy was assessed using 200 μM monodansylcadaverine (MDC, Sigma, USA) staining for 60 min at 27 °C and 200 nM Lyso-Tracker Red DND-99 (Life technologies, Carlsbad, CA, USA) staining for 30 min at 27 °C. Confocal images were captured using an FV1000 confocal microscope (Olympus, Tokyo, Japan).

### 2.7. Statistical Analysis

Statistical differences were evaluated by Student’s *t*-test for unpaired samples. The level of statistically significant difference was set at * *p* value < 0.05, ** *p* value < 0.01, and *** *p* value < 0.001.

## 3. Results

### 3.1. Construction of dCas9 and sgRNA Expression System

**Figure 1:**
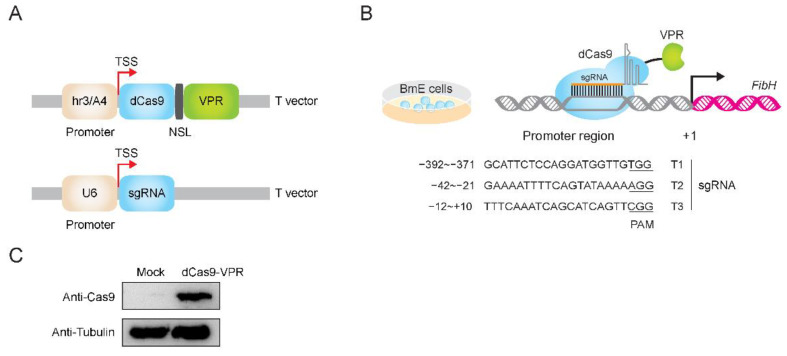
CRISPRa system in BmE cells. (**A**) CRISPR/dCas9 gene activation system. The codon-optimized dCas9 was initiated with hr3/A4 promoter. A nuclear localization signal (NLS) and a tripartite activator (VPR; composed of VP64, p65, and Rta) were fused to the C terminal of dCas9. (**B**) Single guide RNA (sgRNA) target site in *the FibH* promoter region. The sgRNA scaffold was under the control of a U6 promoter. TSS, transcription start site; BmE, *Bombyx mori* embryonic cells. (**C**) Western blot analysis of dCas9-VPR protein. The VPR activation domains was fused to dCas9 and tubulin was used as control.

The nuclease-null dCas9 protein was obtained by mutating spCas9 protein at two sites, D10A and H840A [17]. In a previous study, we tested both CRISPR activation and CRISPR interference vector system in silkworm cells [19,35]. As shown in Figure 1A, the codon-optimized dCas9 with a nuclear localization signal (NLS) and a tripartite activator (VPR; composed of VP64, p65, and Rta) at the C-terminus was driven by the promoter hr3/A4. We designed three single guide RNAs (sgRNAs) targeting the early upstream of the *FibH* gene which followed the GN_(19)_NGG sgRNA designing principle [11,12,36,37], and then the sgRNAs were cloned into the pUC57-U6-sgRNA vector. In this vector, U6 promoter was used to initiate the transcription of sgRNA scaffold (Figure 1B). The expression of dCas9-VPR in BmE cells at 48 h after transfection was confirmed using Western blot (Figure 1C), suggesting that the dCas9-VPR protein was successfully translated in BmE cells.

### 3.2. The Endogenous FibH Gene of BmE Cells Was Activated by CRISPRa System

**Figure 2:**
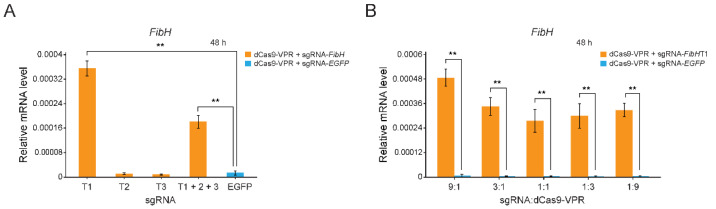
Transcriptional activation of *FibH* gene in BmE cells. (**A**) Relative mRNA levels of *FibH* gene at the 48 h after transient transfection of the combination of dCas9-VPR with sgRNA-*FibH*T1, sgRNA-*FibH*T2, sgRNA-*FibH*T3, sgRNA-*FibH*T1 + 2 + 3, and sgRNA-*EGFP*, respectively. (**B**) Relative mRNA levels of *FibH* gene at the 48 h after transient transfection of a combination of sgRNA-*FibH*T1 with dCas9-VPR in the transfection ratios of 9:1, 3:1, 1:1, 1:3, and 1:9, respectively. Expression of the silkworm housekeeping gene *ribosomal protein L3* (*Rpl3*) was used as a control. Values are means ± SD (*n* = 3). Asterisks represent significant differences determined by a Student’s *t*-test at *p* value < 0.01 (**).

The transcription of *FibH* gene was extremely low in normal BmE cells (Figure 2A). To activate the *FibH* gene’s expression, we first designed three sgRNAs for the transcription start site (TSS) of *FibH* gene, where sgRNA-*FibH*T1, sgRNA-*FibH*T2, and sgRNA-*FibH*T3 were located at −392 to −370, −42 to −21, and −12 to +10 positions, respectively (Table S1). Then, two plasmids (dCas9-VPR vector and one of the sgRNA vectors) were co-transfected into BmE cells, and we found that sgRNA-*FibH*T1 exhibited the highest target gene (*FibH*) activation effect compared with sgRNA-*FibH*T2, sgRNA-*FibH*T3, and sgRNA-*EGFP* (negative control) on the basis of dCas9-VPR expression (Figure 2A). Next, BmE cell were transfected with different ratio of sgRNA and dCas9-VPR (9:1, 3:1, 1:1, 1:3, and 1:9), and the *FibH* expression increased to the maximum level at the ratio of 9:1 (Figure 2B).

### 3.3. Cellular Stress Responses-Related Genes Were Significantly Up-Regulated

**Figure 3:**
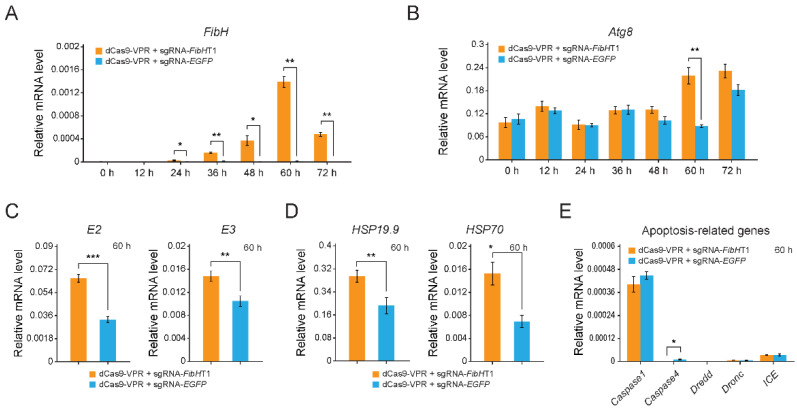
Expression profiles of *FibH* and cellular stress responses-related genes. (**A**,**B**) Relative mRNA levels of *FibH* (**A**) and *Atg8* (**B**) after transient transfection of a combination of sgRNA-*FibH*T1 with dCas9-VPR in the transfection ratio of 9:1, every 12 over 72 h. (**C**–**E**) Relative mRNA levels of ubiquitin-proteasome system-related genes (**C**), heat shock response-related genes (**D**), and apoptosis-related genes (**E**) at the 60 h in *FibH-*activated BmE cells. Expression of the silkworm housekeeping gene *ribosomal protein L3* (*Rpl3*) was used as a control. Values are means ± SD (*n* = 3). Asterisks represent significant differences determined by a Student’s *t*-test at *p* value < 0.05 (*), *p* value < 0.01 (**), and *p* value < 0.001 (***).

Firstly, we investigated the time-course transcriptional profiling of the *FibH* gene after transient transfection of sgRNA-*FibH*T1 and dCas9-VPR at the ratio of 9:1, every 12 h over 72 h. Sixty hours after transfection, the *FibH* expression level increased to maximum, which is about 160 times that of the control level (Figure 3A). In previous study, we found that secretion-deficient and high-molecular-weight *FibH* was able to perturb cellular homeostasis [31,32], which might actuate cellular stress responses, such as autophagy, ubiquitin-proteasome proteolysis and heat shock response [38]. Next, we investigated the mRNA levels of autophagy gene (*Atg8*, a marker gene of autophagic activity [39]), ubiquitin-proteasome system-related genes (*E2* and *E3*), heat shock response-related genes (*Hsp19.9* and *Hsp70*), and apoptosis-related genes (*Caspase1*, *Caspase4*, *Dredd*, *Dronc*, and *ICE*) at 60 h after transfection by qRT-PCR. As shown in Figure 3B, the mRNA levels of the *Atg8* exhibit a tendency of increase from 48 to 72 h, and showed the most significant difference at the 60 h, the same time when *FibH* expression level increased to a maximum, in *FibH*-activated BmE cells. Consistent with this, the mRNA levels of *E2*, *E3*, *Hsp19.9* and *Hsp70* were also up-regulated (Figure 3C,D). However, none of apoptosis-related genes was up-regulated in *FibH*-activated BmE cells (Figure 3E).

Meanwhile, we activated expression of another endogenous fibroin gene (*P25*) by CRISPRa system, when the mRNA level of *P25* increased to over 500-fold compared to control (125-fold compared to *FibH* maximum) (Figure S1A), the mRNA levels of *Atg8* showed no significant difference compared to control (Figure S1B), suggesting that activation of *P25*, even at a much higher level of expression than that of *FibH*, did not lead to up-regulation of the marker gene of autophagy *Atg8*.

### 3.4. Autophagy Was Triggered in FibH-Activated BmE Cells

**Figure 4:**
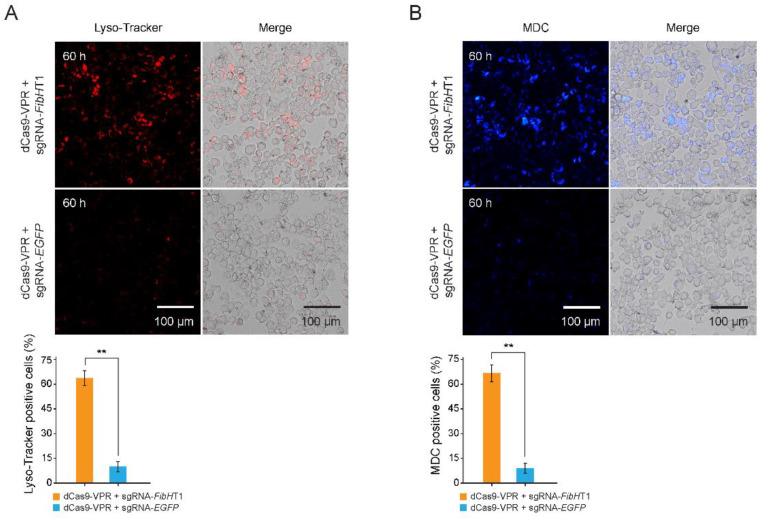
Autophagy assay of *FibH*-activated BmE cells. (**A**,**B**) Lyso-tracker staining (**A**) and the monodansylcadaverine (MDC) staining (**B**) of BmE cells at the 60 h after the condition of transient transfection of a combination of sgRNA-*FibH*T1 with dcas9-VPR in the transfection ratio of 9:1. The lysosome is signaled in red. The autophagic cell is signaled in blue. Values are means ± SD (*n* = 3). Asterisks represent significant difference determined by Student’s *t*-test at *p* value < 0.01 (**).

To confirm whether the activated *FibH* gene triggered cellular stress responses, we performed autophagy assay through cell staining. The *FibH*-activated BmE cells were positively stained red by lyso-tracker red (a dye used to label acidic lysosomes) (Figure 4A) and were also positively stained blue using monodansylcadaverine (MDC, a compound used to label acidic endosomes, lysosomes and autophagosomes) (Figure 4B). Both staining results show that autophagy occurred in more than 60% of the cells after *FibH* activation, but less than 10% in the control. These results suggest that the autophagy was strongly triggered in the *FibH*-activated BmE cells.

## 4. Discussion

The development of silk gland cells and regulation of the fibroin gene are two crucial issues in silkworm studies [21,22,23]. *B. mori* fibroin-deficient mutants are well-known genetic resources to study the silk production process [28,29,31,32]. Deficient fibroin proteins caused by fibroin gene (*FibH* or *FibL*) variations in these mutants interrupt the silk production process [27,28,29,32]. To understand how deficient fibroin proteins regulate the downstream factors and their biological functions in the development of silk gland cells, we constructed a CRISPRa system to activate the transcription of endogenous *FibH* gene of BmE cells. We found that the cellular stress response-related genes and autophagy were triggered when *FibH* was activated.

The VPR activating domain used in the study has been reported to have great potential for its use in regulating gene expression in BmE cells and silkworm individuals [19]. We are the first to use dCas9-VPR to activate *FibH* gene expression in BmE cells, and we found a condition of transfection (sgRNA and dCas9-VPR vectors with a 9:1 molar ratio; cell culture for 60 h) for maximizing target gene expression (Figure 2A,B and Figure 3A). We proposed that the reason causing this unequal transfection ratio of sgRNA: dCas9 might be the difference of driving efficiency between the hr3/A4 and U6 promoters in BmE cells.

Previous studies of silks revealed that terminal domains are highly important for the assembly of silk proteins [40,41,42]. The *FibH* gene, encoding a high-molecular-weight (350 kDa) structural protein of fibroin with no catalytic or regulatory function, has a tissue-specific expression pattern in PSG cells and is completely closed in non-silk gland cells [43]. Once the fibroin fails to form the fibroin unit (FibH-FibL-P25 complex), it cannot be secreted out of the cells, which leads to the PSG atrophy [26,30]. The mutant/misfolded/aggregated fibroin proteins can perturb cellular homeostasis and endanger the cells under severe stress conditions. Therefore, the stressed cells initiate adaptive responses aiming at reducing damage to the cells, such as a decrease in global protein translation and an increase in proteins of the proteolytic system [38,44,45,46]. These cellular stress responses were also observed in *FibH* knock-out silkworms using CRISPR/Cas9 system [47]. The loss-of-function mutation caused an accumulation of abnormal FibH protein and aroused the activation of proteasomes as well as autophagy process in the silk gland cells [47], which was consistent with our results for the autophagy assay and suggested that accumulation of secretion-deficient (abnormal or mutant) FibH protein in the silk gland cells might lead to more activation of autophagy process to promote the rapid degradation of such abnormal proteins and in cells. Thus, we suspected that the cellular stress responses might be caused by the secretion-deficient fibroin protein that is encoded by the CRISPR-activated *FibH* gene in BmE cells, further raising the possibility that secretion-deficient FibH was responsible for the PSG cell atrophy in fibroin-deficient *B. mori* mutants. Interestingly, none of the genes related to apoptosis were up-regulated in *FibH*-activated BmE cells (Figure 3E). We inferred that there might be two reasons leading to this phenomenon: (i) the amount of *FibH* in BmE cells was not sufficient to induce apoptosis; (ii) the *FibH*-activated BmE cell had not reached the time needed to induce apoptosis. The detailed mechanism needs to be further studied.

Our study showed a significant positive correlation between activated *FibH* gene expression and cellular stress responses. Importantly, this might provide a potential model for studying the autophagy caused by a high-molecular-weight protein and lay a foundation for the understanding of silk gland development in silk-spinning insects.

## Data Availability

Not applicable.

## Supplementary Materials

The following are available online at https://www.mdpi.com/article/10.3390/insects12060552/s1, Table S1: Single guide RNA (sgRNA) sequences, Table S2: Primer sets for qRT-PCR.
