# Supplementary material for: CRISPR-Mediated Endogenous Activation of Fibroin Heavy Chain Gene Triggers Cellular Stress Responses in Bombyx mori Embryonic Cells
Source: Insects. 2021 Jun 13;12(6):552. doi: 10.3390/insects12060552 (PMC8231919; doi:10.3390/insects12060552)
Supplement: Supplementary file 1 [file insects-12-00552-s001.zip › insects-1232828-supplementary.pdf]

Supplementary data

CRISPR-mediated endogenous activation of fibroin heavy chain gene triggers cellular stress responses in *Bombyx mori* embryonic cells

Wenbo Hu [1,2], Xiaogang Wang [1], Sanyuan Ma [1,2], Zhangchuan Peng [1,2], Yang Cao [1,2] and Qingyou Xia [1,2,*]

[1] State Key Laboratory of Silkworm Genome Biology, Biological Science Research Center, Southwest University, Chongqing 400715, China.
[2] Chongqing Key Laboratory of Sericulture Science, Chongqing Engineering and Technology Research Center for Novel Silk Materials, Beibei, Chongqing, China.
* Correspondence: xiaqy@swu.edu.cn;

Table of Contents:

Table S1. Single guide RNA (sgRNA) sequences.

Table S2. Primer sets for qRT-PCR.

Figure S1. Transcriptional activation of *P25* gene in BmE cells

Table S1. Single guide RNA (sgRNA) sequences.

Name	sgRNA sequence (5′ - 3′)	Position in promoter	Strand
*FibH*T1	GCATTCTCCAGGATGGTTGTGG	−392~−371	Sense
*FibH*T2	GAAAATTTTCAGTATAAAAAGG	−42~−21	Sense
*FibH*T3	TTTCAAATCAGCATCAGTTCGG	−12~+10	Sense
P25	AAATAAAATGGCGTCGCCGCGG	−125~−143	Sense
EGFP	AGTTCAGCGTGTCCGGCGAGGG	\	\

The underlined bases represent the protospacer adjacent motif (PAM).

Table S2. Primer sets for qRT-PCR.

Name	Forward (5′ - 3′)	Reverse (5′ - 3′)
FibH	TCTGTGTCATCTGCTTCATCTCG	TATCCAGGACGAAGTAAGAAACAA
P25	AGCCGCTGTGGCAGTTTTG	TAGGTGGCGTTGAAGTATGG
Atg8	GAGAAAGGCTGAGGGCGA	TCGTCGGAAAATGCTATGTAT
E2	ATGGAAACTGAACATTTAGCG	TCAGCAGGAGGAGGTCGT
E3	GCAAACATAACTGAAACATACC	TCCGTGGCTTGGAGACT
Hsp19.9	GGCAAACACGAGGAGAA	TGGGTGCGATTACAGACA
Hsp70	GGGTGGTGGAACCTTTG	GGCACGGTTGTCTTTTCT
Caspase-1	CTTCACTGCTGATAAATGTCC	TTTCTCCAAGAGTAATAACCTG
Caspase-4	GAAATACGCTACGACATACG	ACGACTTCAAAGCCAAACT
Dredd	AGCCGTATGACATGGTATCT	TGTTTCCAAGTGGGTTTC
Dronc	GAGATGGGACACTGGGATTT	GTGGAAGCACGGGATGG
ICE	TATTCGCTGCCGACCAA	CGTAAGACGCCCCTGCT
Rpl3	CGGTGTTGTTGGATACATTGAG	GCTCATCCTGCCATTTCTTACT

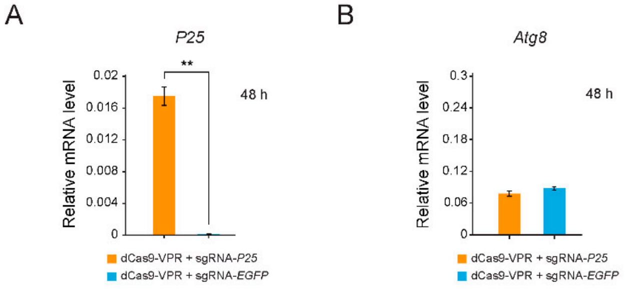

Figure S1. Transcriptional activation of *P25* gene in BmE cells. (**A**,**B**) Relative mRNA levels of *P25* (**A**) and *Atg8* (**B**) at the 48 h after transient transfection of the combination of dCas9-VPR with sgRNA-*P25*. Expression of the silkworm housekeeping gene *ribosomal protein L3* (*Rpl3*)

was used as a control. Values are means ± SD (n = 3). Asterisks represent significant differences determined by a Student's *t*-test at *P* value < 0.01 (**).
